# Spatio-Temporal Evolution of Ecological Network Resilience in the Poyang Lake Eco-Economic Zone from the Perspective of Complex Network Analysis

**DOI:** 10.3390/biology15141136

**Published:** 2026-07-12

**Authors:** Xinyi Huang, Sichen Wei, Wenkai Ding, Yian Xiao, Qitao Su

**Affiliations:** 1 Key Laboratory of Jiangxi Province for Biological Invasion and Biosecurity, School of Life Sciences, Jinggangshan University, Ji’an 343009, China; 2408142040@jgsu.edu.cn (S.W.); 2408143014@jgsu.edu.cn (W.D.); suqitao@jgsu.edu.cn (Q.S.); 2 School of Life Sciences, Jinggangshan University, Ji’an 343009, China; 3 Two Mountains College, Jinggangshan University, Ji’an 343009, China

**Keywords:** ecological network, Poyang Lake Eco-economic Zone, ecological resilience, morphological spatial pattern analysis

## Abstract

Ecological network resilience is an important indicator for evaluating whether regional ecosystems can maintain structural connectivity and functional stability under external disturbance. However, long-term changes in ecological network structure and robustness in the Poyang Lake Eco-economic Zone remain insufficiently understood. Based on land-use data from 1990, 2000, 2010, and 2020, this study integrated Morphological Spatial Pattern Analysis (MSPA), landscape connectivity assessment, the MCR model, complex network analysis, and robustness simulations to evaluate the spatio-temporal evolution of ecological network resilience in the Poyang Lake Eco-economic Zone. The results showed that the area of selected ecological sources in the study area decreased from 39.97% in 1990 to 33.14% in 2020, indicating continuous source-area shrinkage and habitat fragmentation. The ecological resistance surface showed increasing spatial heterogeneity, with relatively low resistance in mountain and lakeside wetland areas and high resistance in the central plain areas affected by agricultural and construction-land expansion. Topological analysis showed that network density increased from 0.10 to 0.15, average path length decreased from 3.04 to 2.53, and network efficiency increased from 0.34 to 0.45, suggesting enhanced local connectivity and transmission efficiency. However, the largest connected component decreased from 37 to 26, indicating a decline in regional-scale connectivity and an increasing risk of network fragmentation. Robustness simulations further showed that the network was relatively tolerant to random disturbance but highly sensitive to the priority failure of key nodes and corridors. Overall, the ecological network evolved from relatively continuous connectivity toward local clustering and residual trunk corridors. These findings suggest that ecological restoration in the Poyang Lake Eco-economic Zone should prioritize major ecological sources, cross-regional corridors, stepping-stone habitats, and critical linkage areas to improve network-level resilience.

## Simple Summary

This study examined changes in ecological network resilience in the Poyang Lake Eco-economic Zone (Jiangxi Province) from 1990 to 2020. The results showed that ecological source areas continued to shrink and become more fragmented, while the network gradually shifted from overall connectivity to local clustering. Although the network was relatively tolerant to random disturbance, it was highly sensitive to the loss of key nodes and corridors. Therefore, ecological restoration should prioritize large habitat patches, critical corridors, and stepping-stone habitats to improve regional ecological network resilience. This work provides a scientific basis for land-use planning and helps ensure the long-term ecological stability of this vital region in the face of rapid urbanization and environmental change.

## 1. Introduction

Ecological security is strictly defined as the capacity of ecosystems to maintain their structural integrity and systemic resilience under disturbances, thereby ensuring the continuous provision of essential ecosystem services [1]. Accelerated industrialization and urbanization, characterized by high-intensity land development, have led to landscape fragmentation and a decline in ecosystem services, intensifying the conflict between economic growth and environmental protection [2,3,4]. As a key intersection for national strategies including the Yangtze River Economic Belt and ecological civilization construction, the ecological health of the Poyang Lake Eco-economic Zone is directly linked to regional ecological security and national food security. Current national mandates emphasize optimizing the “Lake-River-Mountain” integrated ecological shield and constructing an interconnected eco-spatial network [5]. Consequently, identifying ecological sources and constructing security patterns is of urgent practical significance for mastering spatial configuration modes and enhancing ecosystem connectivity and resilience [6,7].

Identifying regional ecological networks based on existing natural habitats is an effective approach to balance the supply and demand of ecosystem services and optimize landscape patterns [8,9,10]. The core logic of this planning framework lies in identifying and protecting critical existing ecological sources, and improving the spatial connectivity among these sources through targeted ecological restoration, instead of relying on the artificial reconstruction of complex ecosystems. The concept originated from landscape planning in the 1860s [11] and has since become a focal point in landscape ecology and geography [12]. International research matured early, focusing on model construction, biological population networks, and greenway engineering [13,14,15,16]. While research in China started later, significant progress has been made in studying the structure and function of ecological networks across various spatial scales from an ecological security perspective [17,18,19,20]. The stability of these networks depends on both internal organization and external environmental influences [21]. Current scholarship primarily explores network construction and evaluation [22], characteristic analysis and optimization [23], resilience assessment [24], and the optimization of security patterns based on functional evaluations [25].

Research on regional ecological networks typically follows the paradigm of “source identification-resistance surface construction-corridor extraction-network analysis” [8], emphasizing network continuity based on ecological processes [7]. Common identification methods include land-use overlays, MSPA, and ecosystem service function grading. Resistance surfaces are generally constructed through multi-factor comprehensive assessments, while corridors are extracted using the Minimum Cumulative Resistance (MCR) model [26] or Circuit Theory [27]. The MCR model has high computational efficiency and produces clear and intuitive outputs. It requires only two types of input data, namely ecological sources and a resistance surface, and can effectively extract the main low-resistance species-migration pathways between ecological sources. The model has a relatively simple operational procedure, and its ecological logic is easy to interpret. Moreover, the generated least-cost corridors have clear spatial boundaries, which facilitates their practical application in ecological conservation planning. Therefore, this study selected the MCR model for analysis and used the Linkage Mapper tool to extract ecological corridors for multi-period datasets in batches.

Regarding resilience, ecosystem resilience refers to the capacity of a system to absorb disturbances and reorganize while maintaining its original functions [28,29]. Ecological network resilience serves as a critical indicator for evaluating this capacity [30]. Currently, many studies utilize complex network theory and robustness models to analyze network resilience under “random” and “targeted” attack scenarios, such as calculating connectivity and recovery robustness to parse resilience changes [31] or using structural robustness indices to compare optimization effects [32]. This study focuses on a complex region characterized by the coexistence of large lake-wetland systems and rapid urbanization. By integrating ecological network identification with topological resilience assessment, this study moves beyond the conventional single framework of “ecological source–ecological corridor identification.” Specifically, this study combines MSPA, landscape connectivity assessment, and the MCR model, while further introducing complex network indicators to conduct resilience simulations. This analytical framework can help determine whether the evolution of an ecological network represents a substantive improvement in overall regional connectivity, or merely the formation of locally connected clusters that may increase the risk of landscape fragmentation at the regional scale.

Using the Poyang Lake Eco-economic Zone as an empirical case, this study established two simulated scenarios, namely random attack and targeted attack, to identify the key ecological nodes and corridors that maintain network resilience. It further reveals the spatio-temporal evolution mechanisms of regional ecological network resilience from 1990 to 2020, and provides a transferable methodological framework for ecological restoration and landscape planning in similar lake-wetland and urbanizing regions.

## 2. Materials and Methods

### 2.1. Study Area

**Figure 1:**
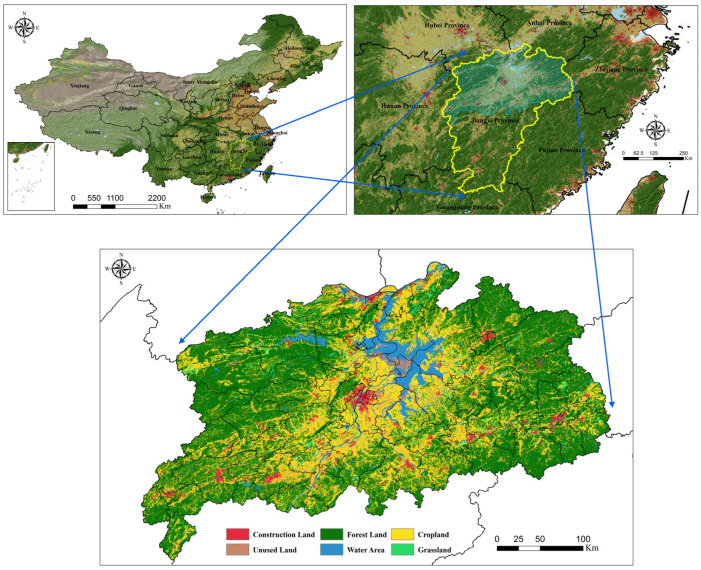
Overview of the Study Area.

The Poyang Lake Eco-economic Zone is located in northern Jiangxi Province, China, between 27°32′–29°45′ N and 115°01′–117°34′ E, covering approximately 92,295.31 km^2^ (Figure 1). In this study, the term “Eco-economic Zone” refers to a regional planning unit centered on Poyang Lake, where ecological conservation, wetland protection, agricultural production, and socioeconomic development are jointly considered. The region contains lakeside plains, hills, mountains, dense river networks, and the Poyang Lake wetland ecosystem, which is important for maintaining the aquatic ecological security of the middle and lower reaches of the Yangtze River and protecting migratory bird habitats. However, rapid urbanization, construction-land expansion, and habitat fragmentation have increased pressure on regional ecological connectivity.

### 2.2. Data Sources and Preprocessing

The basic data used in this study included Landsat 4–5 TM (USGS EROS Center, Sioux Falls, SD, USA) remote sensing images for 1990, 2000, and 2010, and Landsat 8 OLI_TIRS images for 2020. The original spatial resolution of all images was 30 m. Image mosaicking, radiometric calibration, and atmospheric correction were conducted using ENVI 5.3. (Exelis Visual Information Solutions, Boulder, CO, USA) Land use was classified into six categories: farmland, woodland, grassland, water bodies, construction land, and other land. The classification accuracy of land use was verified through sample-based validation using Google Images and land-use patch survey data provided by natural resources authorities.

The digital elevation model (DEM), slope, normalized difference vegetation index (NDVI), and meteorological data were obtained from official public data platforms. All spatial data were uniformly projected to the WGS_1984_UTM Zone 50N coordinate system. To ensure spatial consistency among different analytical steps, all raster datasets were converted to the same coordinate system and resampled to a unified spatial resolution of 250 m × 250 m before analysis. Therefore, MSPA, resistance-surface mapping, ecological corridor extraction, and network topological analysis were all conducted based on 250 m × 250 m raster data.

### 2.3. Land-Use Classification Accuracy Assessment

To improve the reliability of the long-term land-use classification results, classification accuracy assessment was conducted separately for each study year. For the four image periods of 1990, 2000, 2010, and 2020, 600 validation samples were selected for each year using a stratified random sampling strategy based on the six land-use categories, namely farmland, woodland, built-up land, grassland, water bodies, and other land. The reference land-use labels of each validation sample were interpreted using Google Earth high-resolution imagery and existing land-use survey data. Producer accuracy, user accuracy, overall accuracy, and the Kappa coefficient were calculated for each year.

Specifically, we assessed land-use classification accuracy independently for each time point, including 1990, 2000, 2010, and 2020. For each year, 600 validation samples were selected using a stratified random sampling strategy based on the six land-use categories. We calculated producer’s accuracy, user’s accuracy, overall accuracy, and the Kappa coefficient for each year. The revised manuscript now reports class-specific PA/UA values for farmland, woodland, built-up land, grassland, water bodies, and other land, as well as the overall accuracy and Kappa coefficient for each time point. As shown in Table 1, the overall land-use classification accuracies for 1990, 2000, 2010, and 2020 were 76.17%, 85.33%, 83.50%, and 86.50%, respectively, which generally meet the requirements of this study.

### 2.4. Research Framework

**Figure 2:**
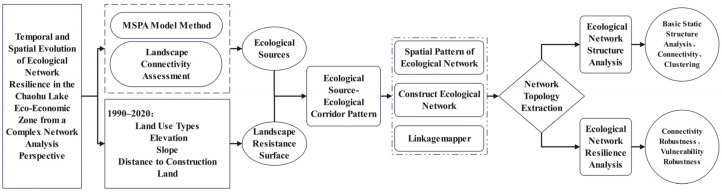
Research Technology Roadmap.

This study focuses on the construction and resilience analysis of the ecological network in the Poyang Lake Eco-economic Zone from 1990 to 2020. Important habitat patches were identified using MSPA, followed by the selection of ecological sources through landscape connectivity evaluations. A comprehensive resistance surface was generated using a weighted index method based on land use, elevation, slope, and distance to construction land. The weights of these factors were determined using the coefficient of variation (CV) method, which objectively reflects the relative spatial dispersion and discriminatory contribution of each factor after standardized reclassification. Ecological corridors were extracted using the Linkage Mapper tool to construct the network. Complex network indices—including degree, degree distribution, degree correlation, average path length, and clustering coefficient—were used to analyze the topological structure. Finally, network robustness models were employed to simulate the evolution of resilience and the impact of core ecological node failure under “random” and “targeted” attack scenarios. The technical roadmap of this study is shown in Figure 2.

### 2.5. Research Methods

#### 2.5.1. Ecological Network Identification

(1) Identification of Ecological Sources

Referring to previous ecological-network studies [33] and considering the regional scale and landscape characteristics of the Poyang Lake Eco-economic Zone, the ten largest MSPA Core patches by area were first selected as candidate ecological sources. This preliminary size-based screening was used because large and continuous Core patches generally provide more stable habitat space and stronger ecological radiation capacity.

The landscape-connectivity importance of these candidate patches was then evaluated using Conefor Sensinode 2.6 (Universidad Politécnica de Madrid, Madrid, Spain) [34,35]. The dispersal distance threshold was set to 2000 m, and the dispersal probability at this distance was set to 0.5 [36,37]. Patch distance was calculated as the edge-to-edge Euclidean distance between habitat patches using Conefor Inputs for ArcGIS 10.2 (Esri, Redlands, CA, USA). Patches with *dPC* ≥ 0.2 were finally identified as ecological sources [38]. The same candidate-patch selection rule, dispersal distance, dispersal probability, patch-distance metric, and *dPC* threshold were applied to all four study years to ensure temporal comparability.

The *PC* was used to characterize the overall level of regional landscape connectivity, whereas the *dPC* was used to measure the relative contribution of each candidate patch to overall landscape connectivity [33,34]. Because the main objective of this step was to identify ecological sources from candidate MSPA Core patches, rather than merely to describe the overall connectivity status of the entire region, *dPC* was used as the core criterion for ecological-source selection. The calculation formula for the *PC* index is:

(1) $$PC=\frac{\sum_{i=1}^{n_{p}}\sum_{j=1}^{n_{p}}a_{i}\cdot a_{j}\cdot p_{ij}}{A_{L}^{2}}$$

(2) $$dPC_{i}=\frac{PC_{full}-PC_{\left\{-i\right\}}}{PC_{full}}\times 100\%$$

In the formula, *n_p_* represents the total number of habitat patches; *a_i_* and *a_j_* denote the areas of patch *i* and patch *j*, respectively (km^2^); *A_L_* is the total area of the study region (km^2^); and *p_ij_* represents the maximum product of all potential dispersal probabilities between patch *i* and patch *j*. *PC_full_* represents the index value of the probability of connectivity when all patches exist in the landscape; *PC*_{*−i*}_ represents the index value of the probability of connectivity for the remaining landscape after patch *i* is removed. The higher the *dPC_i_* value, the higher the importance of patch *i* in the landscape connectivity, and the more significant its status within the landscape.

(2) Ecological Resistance Surface

In this study, the ecological resistance surface was constructed to represent regional-scale potential ecological flow among natural and semi-natural habitat patches, rather than the movement process of a single focal species. Therefore, the selected resistance factors were intended to describe general landscape permeability at the regional scale. Following previous ecological-network and resistance-surface studies [8,39], four factors were selected: land-use type, elevation, slope, and distance from construction land. Land-use type was used to represent the basic habitat attributes and the degree of land transformation. Elevation and slope were used to characterize topographic constraints on ecological movement and landscape permeability. Distance from construction land was used as a proxy for human disturbance intensity because areas closer to built-up land are generally subject to stronger disturbance from urban expansion, infrastructure construction, and human activities.

To ensure temporal consistency among the datasets for the four study periods of 1990, 2000, 2010, and 2020, this study selected resistance factors with complete temporal coverage and stable, consistent spatial resolution. This was intended to reduce subjectivity in the weighting process and to ensure methodological consistency across the four study years. It should be noted that the weights derived from the coefficient of variation method were adopted because they reflect the relative spatial dispersion and spatial discriminatory ability of the standardized factors within the study area. They do not indicate that elevation and slope are absolutely more biologically important than land-use type for all species. This clarification is particularly important because this study focuses on the overall structure and resilience of the regional ecological network rather than the migration behavior of specific species. The weights of all indicators were calculated using the coefficient of variation method, and the specific weighting results are shown in Table 2. The calculation formulas of the coefficient of variation method are as follows:

(3) $$CV_{q}=\frac{\sigma_{q}}{\mu_{q}}\omega_{q}=\frac{CV_{q}}{\sum_{q=1}^{Q}CV_{q}}$$

where *CV_q_* represents the coefficient of variation of the *q*-th resistance factor, and *σ_q_* and *μ_q_* represent the standard deviation and mean value of the *q*-th resistance factor, respectively. *w_q_* represents the weight of the *q*-th resistance factor, and *Q* represents the total number of resistance factors.

(3) Ecological Corridor Extraction

Ecological corridors were extracted using the MCR model and the Linkage Pathways Tool in Linkage Mapper. The MCR model is suitable for identifying potential low-resistance movement pathways between ecological sources based on a resistance surface. In this study, the “Build Network and Map Linkages” module was used to identify neighboring ecological source areas and generate least-cost ecological corridors between them. The MCR model can be expressed as follows:

(4) $$MCR_{s,t}=f_{\min}\sum_{r=1}^{m}\left(l_{r}\times R_{r}\right)$$

where *MCR_s,t_* denotes the minimum cumulative resistance value from ecological source s to landscape unit t; *l_r_* represents the distance or cost-weighted distance of the *r*-th raster unit along a potential migration path; *R_r_* stands for the resistance coefficient of the *r*-th raster unit; *m* is the total number of raster units contained in this path; and *f*_min_ indicates the operation process of screening the path with the minimum cumulative resistance across the entire resistance surface.

After ecological source identification and corridor extraction, the ecological network of each study year was converted into a graph for topological analysis. Each ecological source patch was defined as a network node, and each least-cost ecological corridor connecting two ecological sources was defined as a network edge. Because the extracted corridors represent potential ecological connections between source patches rather than directional movement processes, the graph was constructed as an undirected network.

#### 2.5.2. Analysis of Ecological Network Topological Structure

This study selects structural characteristic indices—including degree distribution, network density, average path length, largest connected component, network efficiency, and average clustering coefficient—from the perspectives of basic static structural characteristics and network robustness to analyze the basic static characteristics, correlation features, and global connectivity of the ecological network in the study area from 1990 to 2020. The specific formulas for these indicators are presented in Table 3.

#### 2.5.3. Analysis of Ecological Network Resilience

The characteristics and degree of an ecosystem’s maintenance of the stability of its structure and functions when subjected to specific disturbances can be measured by robustness [34]. Network robustness measures the stability of ecosystem structures and functions in the face of specific perturbations. Based on complex ecological network theory, connectivity robustness and vulnerability robustness are typically used to evaluate ecological network resilience. This study simulates changes in network resilience after the ecological network is attacked based on a robustness model. The “random attack” scenario involves measuring the changes in network resilience when a certain number of nodes and edges are randomly removed from the ecological network. In this study, the random attack scenario was designed to randomly remove a certain number of nodes and corridors from the ecological network and observe the resulting fluctuations in network resilience. In contrast, the targeted attack scenario removed nodes sequentially according to their degree values, from the highest to the lowest, in order to identify core nodes and key corridors whose failure would cause severe damage to ecological network connectivity.

(1) Connectivity Robustness

Connectivity robustness refers to the capacity of an ecological network to maintain the stability of remaining network element connectivity and to transfer matter and energy after the loss of network elements caused by external factors [36]. Such robustness analysis usually requires assuming a specific scenario. Connectivity robustness is calculated as follows:

(5) $$R_{c}^{\left(t\right)}=\frac{C_{\max}^{\left(t\right)}}{N_{t}}$$

(6) $$C_{\max}^{\left(t\right)}=\max_{S\in C\left(G_{t}\right)}\left|V\left(S\right)\right|N_{t}=N-n_{r}$$

In the formula, $R_{c}^{\left(t\right)}$ represents the connectivity robustness of the ecological network after the t-th disturbance; $G_{t}$ denotes the residual ecological network after disturbance; $C_{max}$ represents the number of nodes contained in the largest connected component of the residual network $G_{t}$; $C(G_{t})$ represents the set of all connected components in $G_{t}$; S denotes any connected component in $C(G_{t})$; V(S) represents the set of nodes contained in component S; $N_{t}$ is the total number of remaining nodes after disturbance; N is the total number of nodes in the original complete ecological network; and $n_{r}$ is the number of nodes removed in the current disturbance.

(2) Vulnerability Robustness

Vulnerability robustness can be used to describe the capacity of an ecological spatial network to maintain the efficient operation of biological flows when subjected to external disturbances; it is typically measured by the global efficiency of the topological network [38]. The higher the global efficiency of the network, the higher the vulnerability robustness. Assuming that energy between nodes always flows along the shortest paths, the calculation formula for vulnerability robustness is:

(7) $$E_{v}^{\left(t\right)}=\frac{2}{N_{t}(N_{t}-1)}\sum_{u<v,u,v\in V_{t}}\frac{1}{d_{uv}^{\left(t\right)}}$$

In the formula, $E_{v}^{\left(t\right)}$ represents the vulnerability robustness of the ecological network after the t-th disturbance; $G_{t}=(V_{t},E_{t})$ denotes the residual ecological network after disturbance, where $V_{t}$ and $E_{t}$ represent the node set and corridor, or edge, set of the residual network, respectively; $N_{t}=|V_{t}|$ is the total number of remaining nodes in the residual network; and $d_{uv}^{\left(t\right)}$ represents the unweighted shortest-path distance between nodes u and v in the residual network $G_{t}$. If no connected path exists between a pair of nodes, then $d_{uv}^{\left(t\right)}=\infty$, and the corresponding value of $1/d_{uv}^{\left(t\right)}$ is defined as 0.

## 3. Results

### 3.1. Landscape Pattern Based on MSPA Method

**Figure 3:**
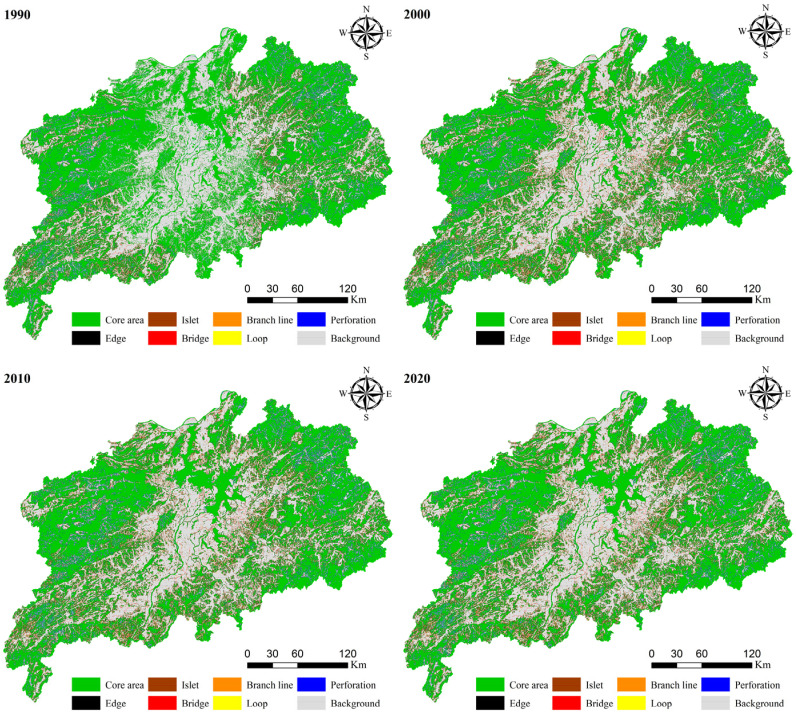
Spatial distribution of MSPA landscape elements.

The MSPA results show clear spatio-temporal changes in landscape elements in the Poyang Lake Eco-economic Zone from 1990 to 2020. Seven landscape types were identified, including Core, Islet, Bridge, Edge, Perforation, Loop, and Branch (Figure 3). Among them, the Core areas mainly correspond to large and relatively continuous natural or semi-natural habitat patches in the Poyang Lake Eco-economic Zone, including woodland-dominated patches in the surrounding mountainous and hilly areas, as well as wetland- and water-associated patches around Poyang Lake. It should be noted that woodland was not assumed to be universally the natural or optimal habitat type for all locations. The classification of woodland as a core habitat component was determined by comprehensively considering the regional land-use pattern and regional ecological functions. Other landscape types, such as Islets, have smaller areas and a more fragmented spatial distribution. Core areas are large habitat patches within the foreground pixels, which are conducive to biodiversity conservation and can provide extensive habitats for biological species. As shown in Table 4, from 1990 to 2020, the ecological sources in the Poyang Lake Eco-economic Zone were primarily distributed in the mountainous regions of the eastern and western parts of the study area. The area of the Core category decreased by 5342.88 km^2^, and its proportion relative to the entire region dropped from 51.23% to 45.44%, indicating a trend of fragmentation and reduction in species habitats.

### 3.2. Selection of Important Ecological Source Patches

**Figure 4:**
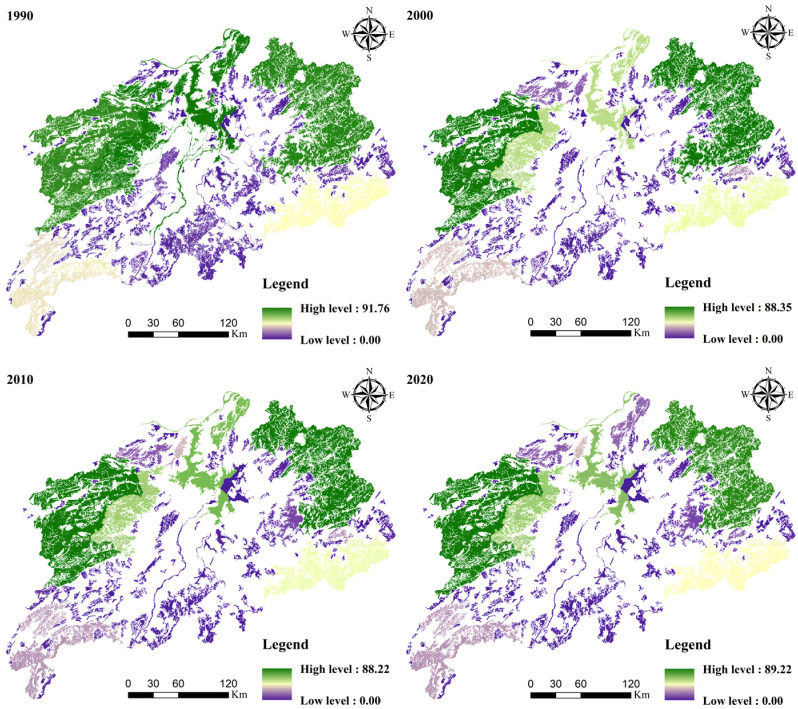
Evaluation of landscape connectivity in the Poyang Lake Eco-economic Zone from 1990 to 2020.

**Figure 5:**
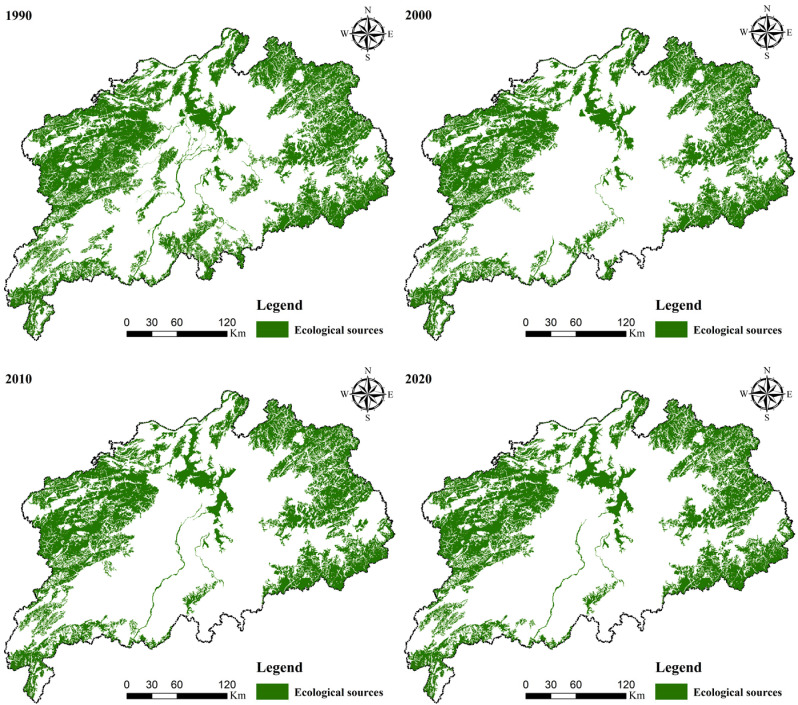
Spatial distribution of ecological sources in the Poyang Lake Eco-economic Zone from 1990 to 2020.

The landscape connectivity results for the Poyang Lake Eco-economic Zone from 1990 to 2020 are presented in Figure 4. The selected ecological sources showed a continuous decreasing trend from 1990 to 2020 (Figure 5). As shown in Table 5, the total area of ecological sources was largest in 1990, reaching 36,889.86 km^2^ and accounting for 39.97% of the study area. By 2020, the source area had decreased to 30,589.04 km^2^, accounting for 33.14% of the study area.

Overall, the ecological sources are primarily distributed in the Huaiyu Mountains in the eastern part of the study area, the Mubu and Jiuling Mountains in the western part, and the Poyang Lake wetland area in the central part. These regions contain numerous large-scale areas of woodland, grassland, and wetlands. In contrast, the inland plain areas have a high degree of intensive land development and lack large-scale land types that meet the screening criteria for ecological sources.

### 3.3. Spatio-Temporal Evolution Characteristics of Ecological Resistance Surface

**Figure 6:**
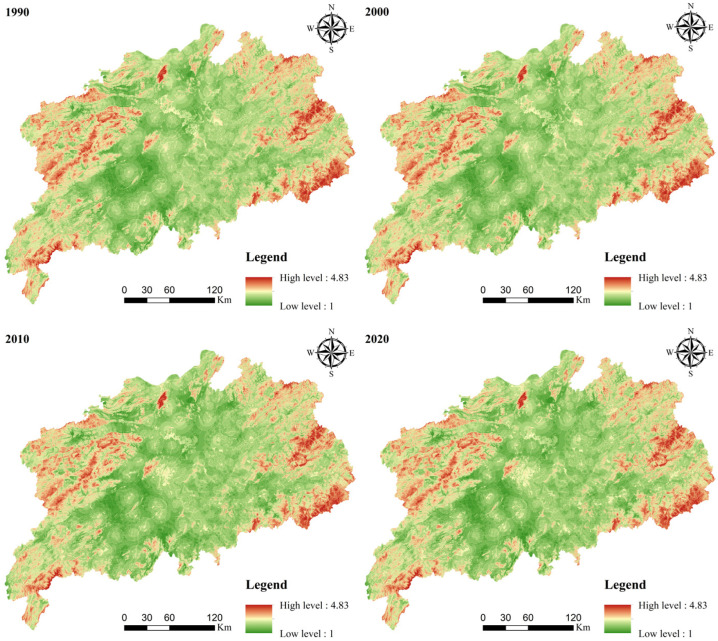
Spatial distribution of ecological resistance values in the Poyang Lake Eco-economic Zone from 1990 to 2020.

From the perspective of spatial distribution, the ecological resistance surface in the study area from 1990 to 2020 exhibited an evolutionary trend characterized by “the shrinkage of core low-resistance areas, the expansion of high-resistance areas, and the enhancement of spatial heterogeneity (Figure 6).” The lakeside wetlands, northern mountains, and eastern hills have maintained low resistance levels over the long term, constituting the core ecological space; meanwhile, the dense urban areas and agricultural reclamation zones in the central plains have formed continuous high-resistance belts, becoming the primary barriers to ecological flow transmission. From the perspective of temporal evolution, the distribution of low-resistance areas was relatively continuous in 1990, with many medium-to-low resistance patches remaining in the central part, resulting in a relatively gentle resistance gradient. Starting from 2000, due to the influence of urbanization and the expansion of construction land, the high-resistance areas in the central region expanded significantly, while the low-resistance areas were continuously squeezed, leading to a gradual increase in the resistance gradient. From 2010 to 2020, the high-resistance areas spread to the surrounding regions, and the medium-to-low resistance patches in the central part basically disappeared, forming a polarized pattern of “low resistance in the north and south, high resistance in the middle,” with spatial heterogeneity continuing to enhance. The spatio-temporal evolution of ecological resistance reflects the continuous disturbance of human activities on the ecological space of the Poyang Lake Eco-economic Zone.

### 3.4. Analysis of the Spatio-Temporal Evolution of the Ecological Network

**Figure 7:**
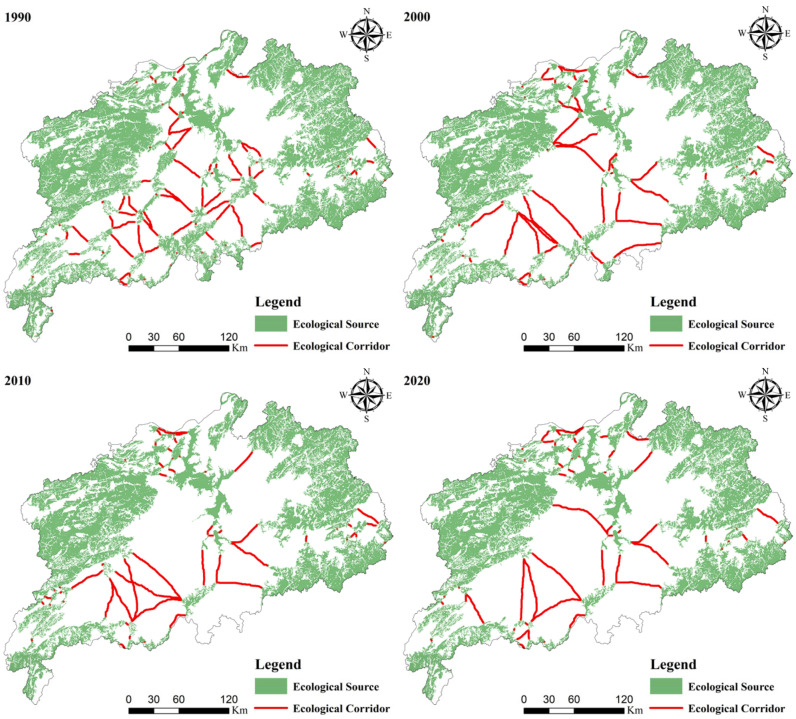
Spatial pattern of ecological sources and corridors in the Poyang Lake Eco-economic Zone from 1990 to 2020.

The construction results of the ecological network in the Poyang Lake Eco-economic Zone from 1990 to 2020 are shown in Figure 7. As can be seen from the figure, the ecological sources in the study area were continuously distributed in 1990, with the northern mountains, southern hills, and lakeside wetlands forming large-scale connected patches. Starting from 2000, the area of sources shrank, continuous patches became fragmented, and the central plains experienced significant recession. By 2020, fragmentation intensified, and the connectivity in the central and southern parts continued to decline, with only the northern mountains, eastern hills, and core lakeside wetlands retaining intact sources, becoming the core carriers of ecological security. Regarding ecological corridors, the network was densely connected in 1990, forming a system with multiple nodes and paths. In 2000, the number and density of corridors decreased, and key corridors in the central part disappeared. By 2010, the network shrunk to a few main trunks. In 2020, the network exhibited characteristics of “trunk-oriented and fragmentation,” with central corridors basically disappearing and only partial connectivity remaining in the northern, southern, and lakeside areas; the overall connectivity significantly decreased, and spatial continuity was severely damaged. It can be concluded that from 1990 to 2020, the ecological network in the study area underwent an evolutionary process of “complete and continuous—gradual fragmentation—residual trunks.” The recession of sources and the fracture of corridors jointly led to a continuous decline in connectivity, reflecting the persistent squeeze of human activities on ecological space. At the same time, it is also evident that the regional ecological security pattern faces severe challenges, necessitating the restoration of network connectivity and integrity through ecological restoration and corridor reconstruction to guarantee the stability and sustainable development of the ecosystem.

### 3.5. Analysis of Ecological Network Topological Characteristics

**Figure 8:**
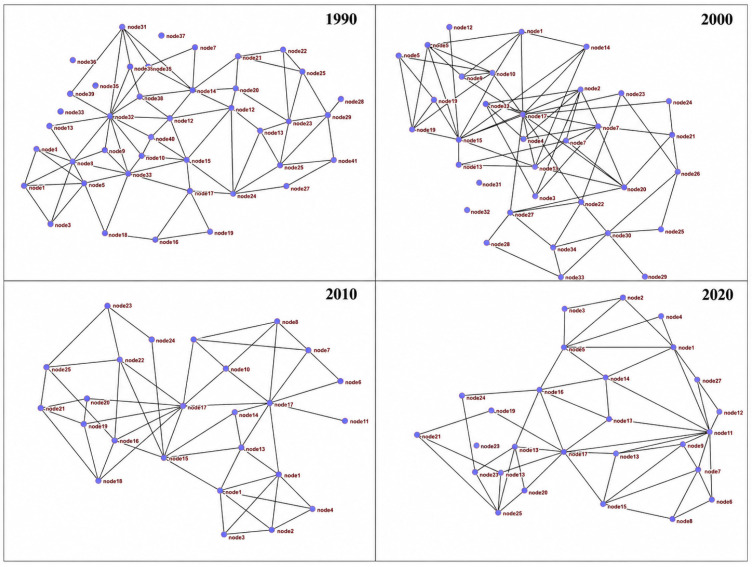
Topological structure of the ecological network in the Poyang Lake Eco-economic Zone from 1990 to 2020.

The topological structure of the ecological network from 1990 to 2020 is shown in Figure 8, and the changes in network indicators are summarized in Table 6. During the study period, the ecological network exhibited phased changes in connectivity, transmission efficiency, and structural stability. The average degree remained relatively stable at approximately 4.0, while network density increased from 0.10 in 1990 to 0.15 in 2020, indicating closer local connections among nodes. The average path length decreased from 3.04 to 2.53, and network efficiency increased from 0.34 to 0.45, suggesting improved local transmission efficiency. However, the largest connected component decreased from 37 in 1990 to 26 in 2020, indicating a decline in global connectivity and an increasing risk of network fragmentation. Overall, from 1990 to 2020, the ecological network of the Poyang Lake Eco-economic Zone exhibited structural evolution characteristics of “improved connection density, enhanced transmission efficiency, and declined global connectivity,” which also reflects that under human activity and interference, the ecological network has gradually evolved from integral connectivity toward local clustering and residual trunks.

### 3.6. Resilience of the Ecological Network in the Poyang Lake Eco-Economic Zone

#### 3.6.1. Connectivity Robustness

**Figure 9:**
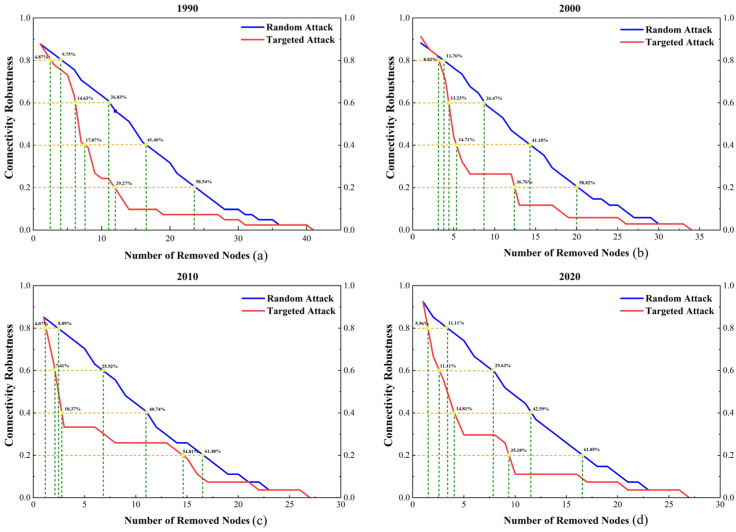
Simulation results of ecological network connectivity robustness in the Poyang Lake Eco-economic Zone from 1990 to 2020.

The connectivity robustness of the ecological network showed clear differences between random attack and targeted attack scenarios from 1990 to 2020 (Figure 9). Under random attack, the robustness curves declined relatively slowly, indicating that the network had a certain degree of structural redundancy against random node loss. In contrast, under targeted attack, the robustness curves dropped rapidly during the early stage of node removal, suggesting that the overall connectivity of the ecological network depended strongly on a limited number of important nodes.

From a temporal perspective, the network showed relatively stable and slightly improved robustness under random attack. When connectivity robustness declined to 0.2, the required node-removal ratio increased from 58.54% in 1990 to 58.82% in 2000, 61.48% in 2010, and 61.85% in 2020. This indicates that the ecological network maintained a relatively strong ability to resist random disturbance throughout the study period, and its residual connectivity under random node loss was slightly enhanced in the later period.

However, the network remained highly vulnerable to targeted attack. In 2010, connectivity robustness declined to 0.6 and 0.4 after removing only 7.41% and 10.37% of key nodes, respectively, showing the strongest early-stage sensitivity. By 2020, although the network showed improved tolerance to random disturbance, targeted removal of only 11.11% and 14.81% of key nodes still reduced robustness to 0.6 and 0.4, respectively. Overall, the ecological network became slightly more tolerant to random disturbance from 1990 to 2020, but its dependence on critical nodes and corridors remained the main weakness of regional ecological connectivity.

#### 3.6.2. Vulnerability Robustness

**Figure 10:**
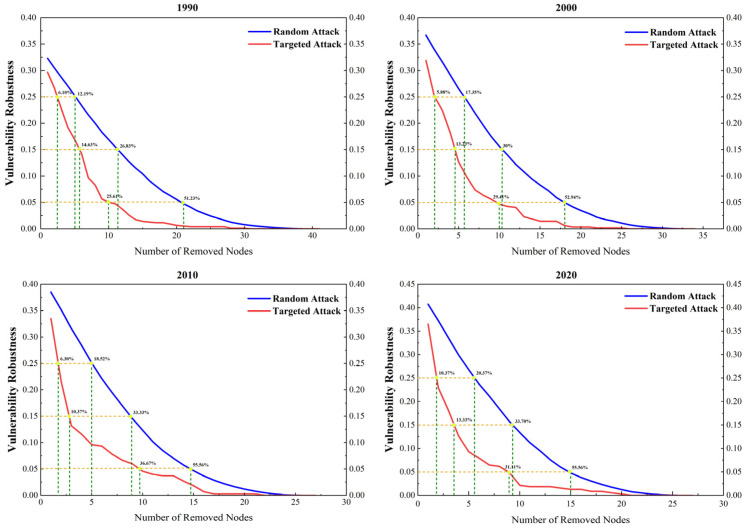
Simulation results of ecological network vulnerability robustness in the Poyang Lake Eco-economic Zone from 1990 to 2020.

The vulnerability robustness of the ecological network showed different response patterns under random attack and targeted attack scenarios from 1990 to 2020 (Figure 10). Under random attack, the robustness curves declined gradually, indicating that the network could maintain a certain level of transmission efficiency after random node loss. In contrast, under targeted attack, the curves dropped sharply in the early stage, suggesting that the removal of important nodes rapidly weakened the operational efficiency of the ecological network.

From a temporal perspective, the network showed a gradual improvement in vulnerability robustness under random attack. When vulnerability robustness declined to 0.25, 0.15, and 0.05, the required node-removal ratios increased from 12.19%, 26.83%, and 51.23% in 1990 to 20.37%, 33.70%, and 55.56% in 2020, respectively. This indicates that the ecological network became more capable of maintaining residual transmission efficiency under random disturbance during the study period.

However, the network remained more sensitive to targeted attack. In 1990, vulnerability robustness declined to 0.25, 0.15, and 0.05 after removing only 6.10%, 14.63%, and 25.61% of key nodes, respectively. By 2020, the corresponding removal ratios increased to 10.37%, 13.33%, and 31.11%, showing a limited improvement in resistance to key-node loss. In summary, from 1990 to 2020, the ecological network showed gradually enhanced vulnerability robustness under random attacks. Although its robustness under targeted attacks also improved to some extent, the network remained clearly vulnerable, indicating that key nodes still played an important role in maintaining network transmission efficiency.

## 4. Discussion

### 4.1. Methodological Considerations and Uncertainties

This study integrates the MSPA method, landscape connectivity assessment, the MCR model, complex network analysis, and robustness simulation to evaluate the spatio-temporal evolution of ecological network resilience in the Poyang Lake Eco-economic Zone. Compared with a single landscape pattern index, this integrated framework links four key analytical steps: habitat patch identification, potential ecological flow assessment, network topological analysis, and disturbance-response evaluation.

MSPA was used to identify habitat patches with important structural significance. Landscape connectivity indices were then applied to screen ecological sources with high contributions to regional connectivity. Ecological corridors were identified based on the MCR model [40,41]. Finally, complex network indicators and robustness simulations provided quantitative support for evaluating the stability and vulnerability of the ecological network [42,43,44].

However, this framework still contains several uncertainties. First, the resistance surface in this study was constructed only using land-use type, elevation, slope, and distance from construction land. Although these factors can represent the major spatial constraints on ecological flow, they cannot fully capture habitat quality, species-specific dispersal behavior, seasonal hydrological fluctuations, or biological interactions among species. Second, MSPA and least-cost paths can mainly characterize structural connectivity and potential functional connectivity. Actual species movement and gene flow still require further validation using field observations or species-distribution data. In addition, the random attack and targeted attack scenarios used in the robustness analysis were only hypothetical structural stress tests and cannot directly predict the actual occurrence or development of real disturbance events.

Therefore, the results of this study are more suitable for identifying relatively vulnerable areas, critical nodes, and ecological restoration priority areas at the regional scale, rather than for precisely predicting actual ecosystem response processes.

### 4.2. Comparison of the Main Findings with Recent Landscape-Ecology Studies

One of the main findings of this study is that ecological source areas in the Poyang Lake Eco-economic Zone showed a trend of area reduction and increasing fragmentation from 1990 to 2020. This indicates that large natural and semi-natural habitat patches have been under increasing pressure. This pattern is consistent with recent landscape-ecology studies showing that rapid urbanization, industrial application, and infrastructure expansion can disrupt habitat connectivity and increase ecological resistance [45]. In this study, ecological sources were mainly distributed in the Huaiyu Mountains, the Mufu–Jiuling mountainous area, and the Poyang Lake wetland region, suggesting that mountain forests, lakeside wetlands, grasslands, and water-associated habitats jointly support the regional ecological network.

Second, the connectivity pattern of the ecological network in the study area gradually shifted from relatively continuous connectivity to a more fragmented structure characterized by local clustering and remaining trunk corridors. Although network density and local connection intensity increased during some periods, the size of the largest connected component continued to decline. This indicates that improved local connectivity does not necessarily represent an improvement in overall regional connectivity. This finding is of important reference value, as it suggests that ecological network evaluation should distinguish between local connectivity optimization and overall network integrity. Similar studies based on ecological network resilience and complex network theory have also shown that regional-scale vulnerability may increase even when local network structure improves [42].

The ecological network resilience assessment further showed that the network had relatively strong tolerance to random disturbance, but was highly vulnerable to the priority failure of key nodes and core corridors. This pattern indicates that a small number of critical ecological elements play a decisive role in maintaining overall connectivity. In recent years, similar disturbance-scenario simulation methods have been widely used in ecological network studies to identify key nodes, ecological corridors, priority restoration spaces, and network vulnerability areas [42,43].

The results of this study can also provide useful references for territorial ecological restoration planning in the study area. Restoration priority areas can be identified according to the spatial distribution of ecological sources, least-cost corridors, and vulnerable network components. Specifically, the Huaiyu Mountains, the Mufu–Jiuling mountainous area, and the Poyang Lake wetland region should be regarded as primary core conservation zones. Corridors connecting mountain forest habitats with lakeside wetland habitats should be prioritized for protection, while high-resistance corridor sections in plain areas affected by agricultural expansion and construction-land expansion should be treated as key restoration areas. The robustness simulations confirmed that the network was more sensitive to the loss of key nodes. Therefore, ecological sources with high connectivity contribution and their associated corridors should be considered core management objects. Ecological restoration should not only protect large source patches, but also improve secondary ecological corridors and stepping-stone habitats around fragmented sources and weakened corridor sections. Particular attention should be paid to maintaining critical nodes and corridors within the largest connected component, so as to alleviate network fragmentation and enhance ecological network resilience.

### 4.3. International Relevance and Transferability of the Study

Although this study is based on a single regional case, its findings have broader reference value for the field of landscape ecology. Large lake-wetland regions worldwide commonly face similar conflicts among urban expansion, agricultural production, wetland conservation, and biodiversity protection. The Poyang Lake Eco-economic Zone represents a typical coupled lake-wetland-urbanizing landscape, where ecological sources, hydrological corridors, and human land-use pressures interact strongly. Therefore, this case can provide methodological and theoretical references for other floodplain, wetland, and river-basin regions.

The empirical values obtained in this study, such as ecological source area, network density, and robustness thresholds, are specific to the study area and should not be directly generalized to other regions. However, the overall analytical framework is transferable. Specifically, the integrated approach combining Morphological Spatial Pattern Analysis (MSPA), the MCR model, complex network indicators, and robustness simulation can be repeatedly applied to determine whether an ecological network is maintained by numerous redundant local connections or supported mainly by a few highly vulnerable key corridors.

This distinction is important for the theoretical development of landscape ecology because it links spatial pattern evolution with network resilience. It helps explain a phenomenon in which an ecological network may appear to have good local connectivity while becoming increasingly fragile at the overall regional scale. From a practical perspective, the results suggest that ecological planning should not only protect large habitat patches, but also maintain cross-regional corridors and stepping-stone habitats, thereby safeguarding the resilience of the entire ecological network system.

### 4.4. Limitations and Future Research Directions

Several limitations in this study should be acknowledged. First, the ecological network constructed in this study was mainly based on land-use data, MSPA (morphological spatial pattern analysis), the MCR model, and topological indicators. Although these methods are useful for identifying structural connectivity, they cannot fully represent species-specific dispersal behavior, habitat quality, or ecological processes. Second, the robustness simulations were based on hypothetical random disturbance and targeted attack scenarios. Therefore, the results should be regarded only as structural stress tests and should not be directly interpreted as predictions of real disturbance events. Third, this study discussed relevant ecological policies as contextual background, but did not include a policy evaluation design, control region, counterfactual framework, or causal attribution model.

Future research should integrate field survey data, high-resolution remote sensing images, habitat quality assessment, and species-specific movement parameters to construct weighted and dynamic ecological networks. In addition, future studies could combine climate-change scenarios, urban-expansion simulations, and policy implementation data to evaluate how different environmental and management factors influence ecological network resilience. The findings obtained from such work would help provide more precise guidance for ecological restoration, corridor optimization, and territorial spatial planning in the Poyang Lake Eco-economic Zone.

## 5. Conclusions

This study evaluated the spatio-temporal evolution of ecological network resilience in the Poyang Lake Eco-economic Zone from 1990 to 2020 by integrating the MSPA method, landscape connectivity assessment, the MCR model, complex network analysis, and robustness simulations. The results showed that ecological source areas continuously shrank and became increasingly fragmented, decreasing from 36,889.86 km^2^ in 1990 to 30,589.04 km^2^ in 2020. These sources were mainly distributed in the Huaiyu Mountains, the Mufu–Jiuling mountainous area, and the Poyang Lake wetland region.

The ecological resistance surface showed increasing spatial heterogeneity, with low-resistance areas mainly located in mountain and lakeside wetland regions, while high-resistance areas expanded in the central plains affected by industrial application and construction-land expansion. The ecological network gradually shifted from relatively continuous connectivity to local clustering and residual trunk corridors. Although network density and network efficiency increased, the largest connected component declined from 37 to 26, indicating that improved local connectivity did not necessarily represent enhanced regional-scale network integrity.

Robustness simulations showed that the ecological network was relatively tolerant to random disturbance but remained highly sensitive to the priority failure of key nodes and corridors. Therefore, ecological restoration should prioritize not only large ecological source patches, but also ecological corridors, and key linkage areas around fragmented sources and weakened corridor sections. Overall, this study provides a transferable framework for assessing ecological network resilience in lake-wetland and rapidly urbanizing regions, and can support ecological restoration, corridor optimization, and territorial spatial planning in the Poyang Lake Eco-economic Zone and comparable regions.

## Figures and Tables

**Table 1:** Accuracy of land use classification results.

Year	Correct/Total Samples	Farmland PA/UA (%)	Woodland PA/UA (%)	Built-Up Land PA/UA (%)	Grassland PA/UA (%)	Water Bodies PA/UA (%)	Other Land PA/UA (%)	Overall Accuracy (%)	Kappa Coefficient
1990	457/600	82.12/79.43	91.12/88.56	85.12/89.23	84.67/74.56	85.21/83.06	78.01/75.63	76.17	0.72
2000	512/600	94.60/90.46	80.78/86.12	98.99/100.00	80.03/79.94	99.21/100.00	85.23/82.89	85.33	0.82
2010	501/600	90.67/95.00	97.08/93.26	97.64/94.35	85.63/87.75	88.34/98.91	88.45/83.23	83.50	0.81
2020	519/600	88.89/95.34	98.55/92.70	99.53/90.31	90.26/88.45	100.00/97.12	90.56/87.45	86.50	0.83

Note: Producer’s accuracy (PA) represents the ratio of correctly classified samples to the reference samples of a given class, while user’s accuracy (UA) represents the ratio of correctly classified samples to the samples classified as that class. Overall accuracy is used to measure the agreement between the classified samples and the validation samples, and the Kappa coefficient is used to evaluate the classification accuracy.

**Table 2:** Factor resistance value and weight.

Resistance Factor	Weight	Classification Index	Resistance Value
Land-use type	0.24	Construction land	5
		Other land	4
		Farmland and Garden plot	3
		Water body and Grassland	2
		Woodland	1
Elevation/m	0.3	>694	5
		>412~694	4
		>188~412	3
		58~188	2
		<58	1
Slope/(°)	0.29	>20	5
		>12~20	4
		>6~12	3
		2~6	2
		<2	1
Distance from the construction land	0.17	<3000	5
		3000~6000	4
		6000~12,000	3
		12,000~20,000	2
		>20,000	1

**Table 3:** Topological structure analysis indicators for ecological networks.

Goal	Index	Method	Formula	Definition
Network topology	Complex Network Structural Characteristics	Average Degree	$\bar{k}=\frac{1}{N}\sum_{u=1}^{N}k_{u}=\frac{2M}{N}$ $k_{u}=\sum_{v=1}^{N}a_{uv}$	*N* represents the total number of ecological network nodes; *M* represents the total number of ecological corridors; *k_u_* represents the degree of node *u*; *a_uv_* represents the element of the binary adjacency matrix; and $\bar{k}$ represents the average degree of the network.
		Network Density	$\rho =\frac{2M}{N(N-1)}$	*ρ* represents the ecological network density; *N* represents the total number of ecological source nodes in the network; *M* represents the total number of corridors connecting ecological sources.
		Average Path Length	$l=\frac{1}{\left|\Omega \right|}\sum_{\left(u,v\right)\in \Omega }d_{uv}$ $\Omega =\left\{\left(u,v\right)|u<v,d_{uv}<\infty \right\}$	where ℓ denotes the average path length; *d_uv_* is the unweighted shortest path distance between node *u* and node *v*; Ω represents the set of all reachable node pairs, and $\left|\Omega \right|$ is the total number of reachable node pairs within the network.
	Network Robustness	Largest Connected Component	$LCC=\underset{S\in C\left(G\right)}{\max}\left|V\left(S\right)\right|$	*C*(*G*) represents the set of all connected components in network *G*; *S* represents any connected component; *V*(*S*) represents the set of nodes contained in connected component *S*; $\left|V\left(S\right)\right|$ represents the number of nodes in this connected component; *max* indicates the maximum number of nodes among all connected components.
		Network Efficiency	$E_{glob}=\frac{1}{N(N-1)}\sum_{u<v}\frac{1}{d_{uv}}$	*E_glob_* represents the global efficiency of the ecological network; *d_uv_* represents the unweighted shortest-path distance between nodes *u* and; *N* represents the total number of nodes in the network; and the summation range *u* < *v* indicates that all unordered node pairs are traversed without repetition.
		Average Clustering Coefficient	$\bar{C}=\frac{1}{N}\sum_{u=1}^{N}\frac{2e_{u}}{k_{u}(k_{u}-1)}$	where *e_u_* denotes the actual number of edges between neighbor nodes of node *u*, and *k_u_* represents the degree of node *u*. If *k_u_* < 2, the local clustering coefficient of node *u* is set to zero. The average clustering coefficient $\bar{C}$ quantifies the local clustering level of the ecological network.

**Table 4:** MSPA landscape element statistics.

Landscape Type	1990		2000		2010		2020	
	Area /km^2^	The Proportion of the Total Area	Area /km^2^	The Proportion of the Total Area	Area /km^2^	The Proportion of the Total Area	Area /km^2^	The Proportion of the Total Area
Core area	47,284.60	51.23%	42,464.63	46.01%	42,380.43	45.92%	41,941.72	45.44%
Branch line	1072.41	1.16%	2303.82	2.50%	2392.79	2.59%	2449.54	2.65%
Edge	7001.53	7.59%	9022.76	9.78%	8768.06	9.50%	8671.94	9.40%
Perforation	2682.21	2.91%	2750.19	2.98%	2839.50	3.08%	2834.22	3.07%
Islet	114.13	0.12%	478.06	0.52%	481.38	0.52%	491.45	0.53%
Bridge	639.15	0.69%	1441.26	1.56%	1458.39	1.58%	1479.33	1.60%
Loop	291.44	0.32%	611.88	0.66%	583.67	0.63%	598.43	0.65%
Background	33,209.84	35.98%	33,222.71	36.00%	33,391.09	36.18%	33,826.68	36.65%

**Table 5:** Statistics of ecological source area.

Ecological Source	1990		2000		2010		2020	
	Area /km^2^	The Proportion of the Total Area	Area /km^2^	The Proportion of the Total Area	Area /km^2^	The Proportion Of The Total Area	Area /km^2^	The Proportion of the Total Area
	36,889.86	39.97%	31,526.06	34.16%	31,174.29	33.78%	30,589.04	33.14%

**Table 6:** Statistics of ecological network structure characteristics.

Year	Number of Nodes	Number of Edges	Average Degree	Network Density	Average Path Length	Largest Connected Component	Network Efficiency	Average Clustering Coefficient
1990	41	82	4	0.1	3.04	37	0.34	0.46
2000	34	69	4.06	0.12	2.79	32	0.4	0.44
2010	27	54	4	0.15	2.47	25	0.42	0.47
2020	27	54	4	0.15	2.53	26	0.45	0.47

## Data Availability

The publicly available land-use remote-sensing datasets used in this study were obtained from the Geospatial Data Cloud platform (https://www.gscloud.cn/sources/index?pid=263, accessed on 20 March 2026). Socioeconomic statistical data were obtained from the relevant statistical yearbooks of Jiangxi Province. Some original administrative statistical data cannot be made publicly available due to local data confidentiality regulations but are available from the corresponding author upon reasonable request.
